# Recombinant Protein Filovirus Vaccines Protect Cynomolgus Macaques From Ebola, Sudan, and Marburg Viruses

**DOI:** 10.3389/fimmu.2021.703986

**Published:** 2021-08-18

**Authors:** Axel T. Lehrer, Eleanore Chuang, Madhuri Namekar, Caitlin A. Williams, Teri Ann S. Wong, Michael M. Lieberman, Alex Granados, John Misamore, Jake Yalley-Ogunro, Hanne Andersen, Joan B. Geisbert, Krystle N. Agans, Robert W. Cross, Thomas W. Geisbert

**Affiliations:** ^1^Department of Tropical Medicine, Medical Microbiology & Pharmacology, John A. Burns School of Medicine, University of Hawaii at Manoa, Honolulu, HI, United States; ^2^BIOQUAL, Inc., Rockville, MD, United States; ^3^Galveston National Laboratory, Department of Microbiology and Immunology, University of Texas Medical Branch, Galveston, TX, United States

**Keywords:** filovirus, Ebola virus, Marburg virus, Sudan virus, vaccine, preclinical efficacy studies, non-human primates

## Abstract

Ebola (EBOV), Marburg (MARV) and Sudan (SUDV) viruses are the three filoviruses which have caused the most fatalities in humans. Transmission from animals into the human population typically causes outbreaks of limited scale in endemic regions. In contrast, the 2013-16 outbreak in several West African countries claimed more than 11,000 lives revealing the true epidemic potential of filoviruses. This is further emphasized by the difficulty seen with controlling the 2018-2020 outbreak of EBOV in the Democratic Republic of Congo (DRC), despite the availability of two emergency use-approved vaccines and several experimental therapeutics targeting EBOV. Moreover, there are currently no vaccine options to protect against the other epidemic filoviruses. Protection of a monovalent EBOV vaccine against other filoviruses has never been demonstrated in primate challenge studies substantiating a significant void in capability should a MARV or SUDV outbreak of similar magnitude occur. Herein we show progress on developing vaccines based on recombinant filovirus glycoproteins (GP) from EBOV, MARV and SUDV produced using the *Drosophila* S2 platform. The highly purified recombinant subunit vaccines formulated with CoVaccine HT™ adjuvant have not caused any safety concerns (no adverse reactions or clinical chemistry abnormalities) in preclinical testing. Candidate formulations elicit potent immune responses in mice, guinea pigs and non-human primates (NHPs) and consistently produce high antigen-specific IgG titers. Three doses of an EBOV candidate vaccine elicit full protection against lethal EBOV infection in the cynomolgus challenge model while one of four animals infected after only two doses showed delayed onset of Ebola Virus Disease (EVD) and eventually succumbed to infection while the other three animals survived challenge. The monovalent MARV or SUDV vaccine candidates completely protected cynomolgus macaques from infection with lethal doses of MARV or SUDV. It was further demonstrated that combinations of MARV or SUDV with the EBOV vaccine can be formulated yielding bivalent vaccines retaining full efficacy. The recombinant subunit vaccine platform should therefore allow the development of a safe and efficacious multivalent vaccine candidate for protection against Ebola, Marburg and Sudan Virus Disease.

## Introduction

The *Filoviridae* family of viruses includes several highly virulent pathogens, such as *Zaire ebolavirus* (EBOV), *Sudan ebolavirus* (SUDV) and *Marburg marburgvirus* (MARV), which have caused sporadic and large-scale outbreaks in Central and West Africa. These viruses cause hemorrhagic fevers with case-fatality rates up to 90% (1, 2). The world’s second largest EBOV outbreak on record, which took place from 2018-2020 in the Democratic Republic of Congo (DRC), has resulted in 3,481 confirmed cases and 2,299 deaths (3). In November and December 2019, the Ervebo vaccine was approved by the European Medicines Agency and United States Food and Drug Administration, respectively, for the prevention of Ebola Virus Disease (EVD) (4). Ervebo is composed of rVSV-ZEBOV – a recombinant vesicular stomatitis virus where its glycoprotein has been substituted with EBOV surface glycoprotein (GP) to induce immune responses to EBOV (5). While virally vectored vaccines such as Ervebo have shown protective efficacy during experimental use in West Africa and DRC outbreaks (6), we expect that a recombinant subunit vaccine, especially a thermostabilized product (7), would pose less burdensome cold chain requirements for distribution and storage in endemic regions with poor infrastructure. Live virus vaccines are also contraindicated for severely immunocompromised individuals and pregnant women (8) as viral replication of the vaccine strain may induce pathogenicity in vaccinees or in a developing fetus or neonate.

Our group has developed vaccines comprised of adjuvanted recombinant glycoproteins from EBOV, SUDV and MARV that have demonstrated immunogenicity and protective efficacy in mice (9) and guinea pigs (10). In addition, these vaccine formulations have been successfully thermostabilized by lyophilization, resulting in retention of full immunogenicity after several weeks at high temperatures (11). Here we demonstrate immunogenicity and protective efficacy of recombinant protein filovirus vaccines in cynomolgus macaques, the animal model deemed most predictive of vaccine protection in humans.

## Methods

### Antigens

Recombinant subunit proteins were expressed in *Drosophila* S2 cells and purified as previously described (12). In brief, EBOV GP (>90% pure after single-step immunoaffinity purification) was subjected to size exclusion chromatography (SEC) using a Superdex 200 column (GE Healthcare, Piscataway, NJ) equilibrated in phosphate-buffered saline (PBS), pH 7.4. SUDV and MARV GPs were similarly expressed and purified, using antigen-specific monoclonal antibodies for immunoaffinity chromatography followed by SEC. Purified antigens were quality controlled by Coomassie-stained SDS-PAGE to confirm concentration and purity as well as by western-blots probed with antigen-specific monoclonal antibodies to confirm identity. Endotoxin tests performed on several larger bench-scale production lots consistently produced results below detection limit (internal release criteria specify an acceptance level of <25 EU/mg protein).

### Vaccines

Animals were vaccinated intramuscularly with formulations consisting of 25 μg of purified EBOV GP (Mayinga strain), MARV GP (Angola strain), and/or SUDV GP (Gulu strain) combined with 10 mg CoVaccine HT™, a clinical grade proprietary adjuvant provided courtesy of Protherics Medicines Development (London, UK). Antigen dose levels were chosen based on dosing results from rodent studies and adjuvant dose level was selected based on safety data from a previous phase I human clinical trial (NCT01015703). Specific formulations are indicated in the main text for each experiment. Control animals were injected with 10 mg of CoVaccine HT™ in PBS.

### Challenge Viruses

*Zaire ebolavirus* (EBOV) isolate 199510621 (strain Kikwit) originated from a 65-year-old female patient who had died on 5 May 1995 during the Kikwit outbreak in the Democratic Republic of Congo. The study challenge material was from the second Vero E6 passage of EBOV isolate 199510621. Passage 1 of EBOV isolate 199510621 (CDC 807223) was passed once at UTMB by inoculating Vero E6 cells (ATCC CRL-1586) at an MOI of 0.001. Cell culture fluids harvested at day 10 post infection were stored at -80°C as ~1 ml aliquots until used for challenge studies. Deep sequencing indicated that the EBOV population in vials shows greater than 98% 7U (consecutive stretch of 7 uridines). No mycoplasma or endotoxin could be detected (< 0.5 endotoxin units (EU)/ml). *Sudan ebolavirus* (SUDV) isolate 200011676 (strain Gulu) originated from a 35-year-old male patient who had died on 16 October 2000 during the outbreak in Gulu, Uganda. The study challenge material was from the second Vero E6 cell passage of SUDV isolate 200011676. Briefly, Vero E6 cells (ATCC CRL-1586) were inoculated with CDC 808892 (CDC passage 1 of SUDV isolate 200011676) at an MOI of 0.001. Cell supernatants were harvested at day 7 post infection and stored at -80°C as ~ 1 ml aliquots until used. No mycoplasma or endotoxin could be detected (< 0.5 EU/ml). *Marburg marburgvirus* (MARV) Angola isolate 200501379 originated from serum collected on day 17 post onset, 1 day before death from an 8-month-old female patient in Uige, Angola. Study challenge material was from the second Vero E6 cell passage of MARV Angola isolate 200501379. CDC 810820 (Passage 1 material) was inoculated at an MOI of 0.001 onto Vero E6 cells (ATCC CRL-1586). Cell supernatants were subsequently harvested at day 6 post infection and stored at -80°C as ~ 1 ml aliquots until used for challenge. No mycoplasma or endotoxin were detectable (< 0.5 EU/ml).

### Biosafety and Ethics

Non-human primate (NHP) experiments were approved by BIOQUAL, Inc. and University of Texas-Medical Branch (UTMB) Institutional Animal Care and Use Committees (IACUC) and conducted in strict accordance with local, state, federal and institutional policies established by the United States National Institutes of Health. Vaccinations were carried out at BIOQUAL, Inc., and viral challenge occurred at UTMB in their BSL-4 animal facility (Galveston National Laboratory).

### Non-Human Primates and Specimen Collection

To replicate more adequately the population of future vaccine recipients, filovirus-naive, adult cynomolgus macaques (*Macaca fascicularis*) were selected from research-naïve and experienced 4-14 years old animals of both sexes in approximately equal distribution. Animals were randomly assigned to experimental groups and housed at BIOQUAL, Inc. during the pre-challenge vaccination period. Animals were vaccinated intramuscularly with two or three doses of vaccine or only adjuvant at three-week intervals. Serum or plasma was collected periodically until viral challenge at study week 10 after first vaccination.

### Filovirus Challenge

NHP were transferred to UTMB and challenged 10 weeks after first vaccination by intramuscular injection with 1,000 PFU of EBOV (Kikwit strain), 1,000 PFU of SUDV (Gulu strain), or 1,000 PFU of MARV (Angola strain). UTMB study staff was blinded as to the experimental group of each animal and only informed by the Principal Investigator once all challenge control animals had succumbed to disease. Physical exams were conducted, and blood was collected on days 0, 3, 6, 10, 14, 21 and 28 after challenge. NHP were monitored daily and scored for disease progression. Those with marked Filovirus Disease – EVD, SVD, or MVD – were euthanized per UTMB IACUC-approved protocol.

### Luminex Assays

IgG responses were measured in triplicate, utilizing a customized, multiplexed filovirus antigen panel for Luminex-based microsphere immunoassay (MIA) as previously described (13) with the following modifications: pre-challenge serum samples diluted to 1:10,000 were incubated with antigen-coupled microspheres on a shaker for three hours at 37°C. Microspheres were then washed and incubated with goat anti-human IgG, Fcγ fragment-specific antibody conjugated to phycoerythrin (PE) (Jackson ImmunoResearch Laboratories, Inc., West Grove, PA, USA) on a shaker for one hour at 37°C. Median fluorescence intensity (MFI) of the PE signal was quantified by Luminex xPonent 4.2 software on a MAGPIX^®^ system (Luminex Corporation, Austin, TX, USA).

### Hematology and Serum Chemistry

Complete blood counts were assessed, using a laser-based hematologic analyser (Beckman Coulter, Brea, CA, USA). Serum samples were tested for alanine aminotransferase (ALT), aspartate aminotransferase (AST), alkaline phosphatase (ALP), blood urea nitrogen (BUN), creatinine (CRE), and C-reactive protein (CRP) by using a Piccolo point-of-care analyzer and Biochemistry Panel Plus analyzer discs (Abaxis, Inc., Union City, CA, USA).

### Viremia by Plaque Assay

Standard plaque assays were performed, using Vero E6 cell monolayers as previously described (14). Briefly, serial 10-fold dilutions of post-challenge serum or plasma samples were adsorbed to Vero E6 monolayers in duplicate wells of 6-well plates at 37°C for one hour. Following aspiration, monolayers were overlaid with 0.8% SeaPlaque agarose in DMEM, and plates were incubated at 37°C with 5% CO_2_ for 7-10 days to allow plaque development.

### Statistical Analysis

Two-way repeated measures analyses of variance (ANOVAs) were performed using SAS 9.4 (SAS Institute Inc., Cary, NC, USA) to assess differences in immune responses among vaccinated groups and controls, including the interaction between group and study week. Normality assumption of residuals was evaluated and IgG values were log 10 transformed if the assumption was violated. Fisher’s least significant difference post-hoc test was conducted to compare groups at each time point due to small sample size. Kaplan-Meier plots were created to depict vaccinated survivors and log-rank test was conducted to compare vaccinated groups and controls on the survival data. Statistical significance was considered to be reached at a p-value less than 0.05.

## Results

**Figure 1:**
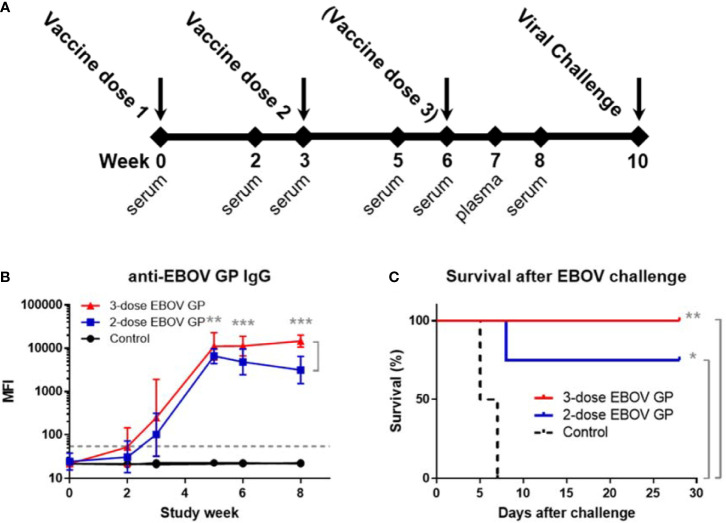
Standard study schedule, immune response to EBOV GP, and survival after EBOV challenge. **(A)** Each study described followed a standard schedule in which cynomolgus macaques were vaccinated at three-week intervals with either two or three doses of vaccine. Controls received adjuvant only. Serum or plasma samples were collected periodically. Animals were challenged with 1,000 PFU of virus at week 10 after initial vaccination. **(B)** Anti-EBOV GP IgG antibodies were measured by MIA at time points relative to first vaccination with EBOV GP. Samples were assayed in triplicate. Each data point for vaccinated groups represents geometric mean ± 95% confidence interval whereas data points for controls were plotted per individual animal. The dashed grey line marks the threshold for positive detection of anti-EBOV GP IgG at 3 standard deviations (SD) above mean MFI of negative control samples. **(C)** Kaplan-Meier survival curves after challenge with EBOV. *p < 0.05, **p < 0.01, ***p < 0.001.

To test the recombinant protein vaccine against EBOV challenge, cynomolgus macaques were administered intramuscular injections with either two doses (n=4) or three doses (n=6) of vaccine containing 25 μg of EBOV GP plus adjuvant (**Figure 1A**). Controls received adjuvant only (n=2). At study week 10 after initial vaccination, animals were challenged intramuscularly with 1,000 plaque-forming units (PFU) of a low-passage, human isolate of EBOV (Kikwit strain). Vaccinated groups showed boosting of EBOV GP-specific IgG responses after the second and third doses of vaccine; however, antibody levels declined from week five to week eight in animals receiving two doses compared to sustained levels until viral challenge among animals receiving a third dose (p<0.01) (**Figure 1B**). All animals administered three doses of vaccine remained completely healthy and survived viral challenge while three out of four animals receiving only two doses of vaccine survived despite the observed decline in IgG titers. Control animals succumbed to Ebola virus disease (EVD) by day 7 after viral challenge (**Figure 1C**). By log-rank test, both vaccine regimens exhibited survival curves significantly different from controls (p=0.018 for two doses and p=0.004 for three doses), however, the difference between two- and three-dose regimens was not statistically significant (p=0.221).

**Figure 2:**
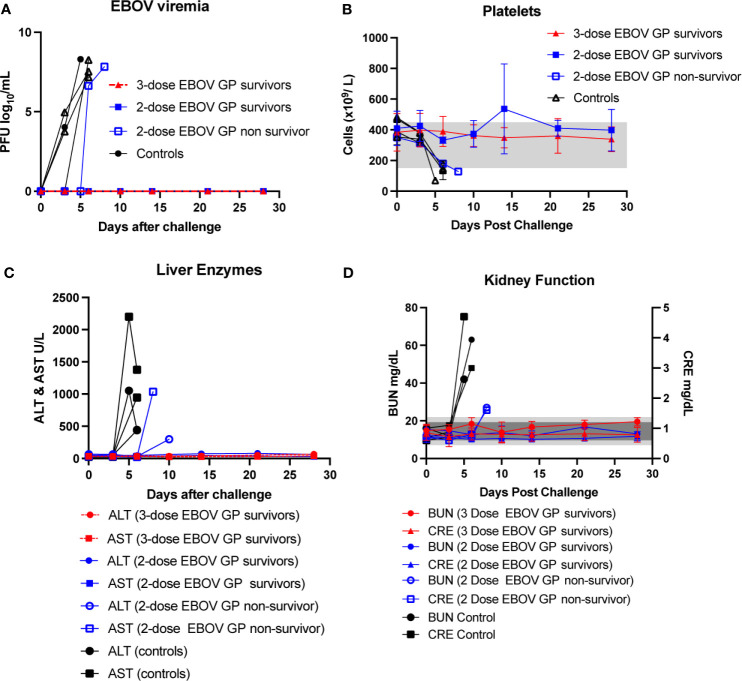
Viremia, platelet counts, liver enzymes, and kidney function after EBOV challenge. **(A)** EBOV viremia as assessed by plaque assay. **(B)** Platelet counts with normal range of 150-450 x 10^3^ cells/μL shown as light grey band. **(C)** Serum ALT with normal range of 10-47 U/L and serum AST with normal range of 11-38 U/L. **(D)** Serum BUN with normal range of 7-22 mg/dL shown as light grey band and CRE with normal range of 0.6-1.2 mg/dL shown as darker grey band. Each data point for vaccinated survivor groups represents mean ± SEM. Data points for controls and the single two-dose non-survivor were plotted per individual animal.

**Figure 3:**
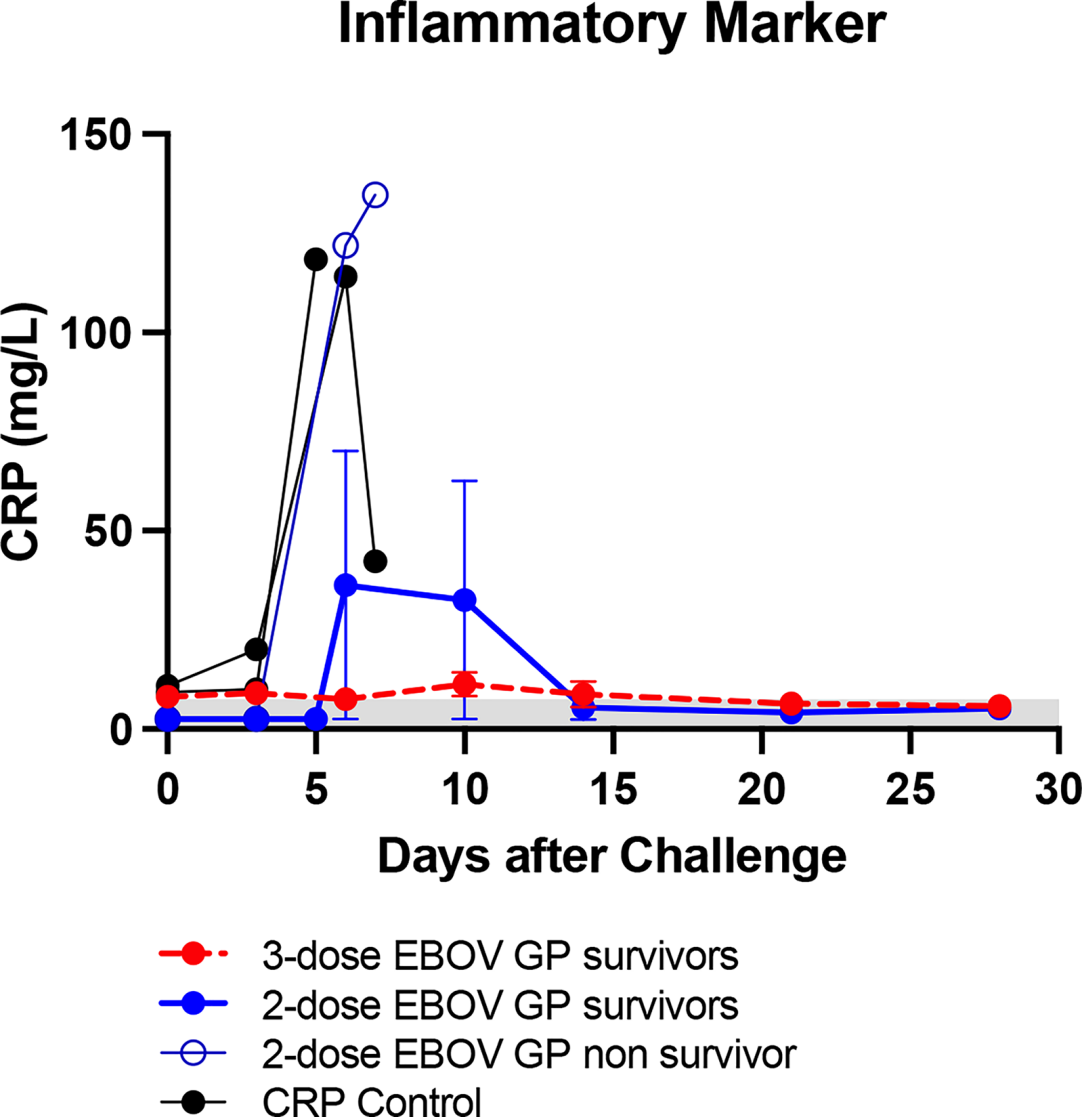
Inflammatory marker after EBOV challenge. Serum CRP with normal range of 0.0-7.5 mg/L shown as light grey band. Each data point for vaccinated groups represents mean ± SEM. Data points for controls and two-dose non-survivor were plotted individually.

After EBOV challenge, vaccinated survivors exhibited no viremia as assessed by plaque assay as well as no change indicative of EVD in platelet counts, serum levels of liver enzymes such as alanine aminotransferase (ALT) and aspartate aminotransferase (AST) and serum markers of kidney function such as blood urea nitrogen (BUN) and creatinine (CRE) (**Figure 2**). In contrast, control animals as well as one out of four two-dose vaccinees succumbed to EVD as manifested by increasing viremia after viral challenge along with a concomitant plunge in platelet counts and surging levels of ALT, AST, BUN and CRE (**Figures 2A–D**). Serum levels of C-reactive protein (CRP) also rose dramatically among the non-survivors (**Figure 3**).

**Figure 4:**
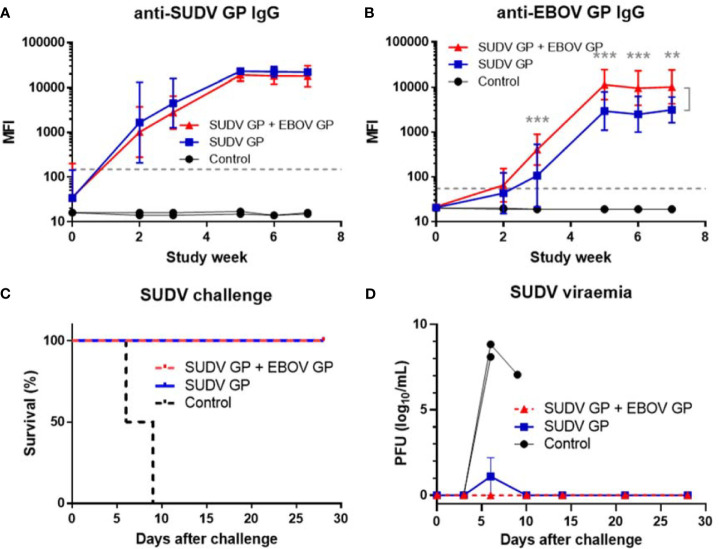
Immune response after SUDV vaccination, survival and viremia after SUDV challenge. MIA performed in triplicate for animals vaccinated at three-week intervals with three doses of SUDV GP with adjuvant (n=4) or SUDV GP + EBOV GP with adjuvant (n=4). Controls received adjuvant only (n=2): **(A)** SUDV GP-binding IgG and **(B)** EBOV GP-binding IgG. Each data point for vaccinated groups represents geometric mean ± 95% confidence interval whereas data points for controls were plotted per individual animal. The dashed grey line marks the threshold for positive detection of antigen-specific IgG at 3 SD above mean MFI of negative control samples. **(C)** Kaplan-Meier survival curves after challenge with SUDV. **(D)** SUDV viremia as assessed by plaque assay. Each data point for vaccinated groups represents mean ± SEM. Data points for controls were plotted per individual animal. **p < 0.01, ***p < 0.001.

In another study, cynomolgus macaques were vaccinated with three doses of either 25 μg of SUDV GP with adjuvant (n=4) or 25 μg of SUDV GP + 25 μg of EBOV GP with adjuvant (n=4) to determine whether different *ebolavirus* GPs can be combined in a single multivalent *ebolavirus* vaccine and retain efficacy. Controls received adjuvant only (n=2). Animals were challenged intramuscularly with 1,000 PFU of a low-passage, human isolate of SUDV (Gulu strain) at week 10 after initial vaccination. SUDV GP-binding IgG rose for all vaccinated animals but did not differ for the two vaccine formulations (p>0.05) (**Figure 4A**). As expected, EBOV GP-binding IgG for the group vaccinated with SUDV GP + EBOV GP was higher than that of the SUDV GP group with significance (p<0.01) observed from week three to week seven (**Figure 4B**).

**Figure 5:**
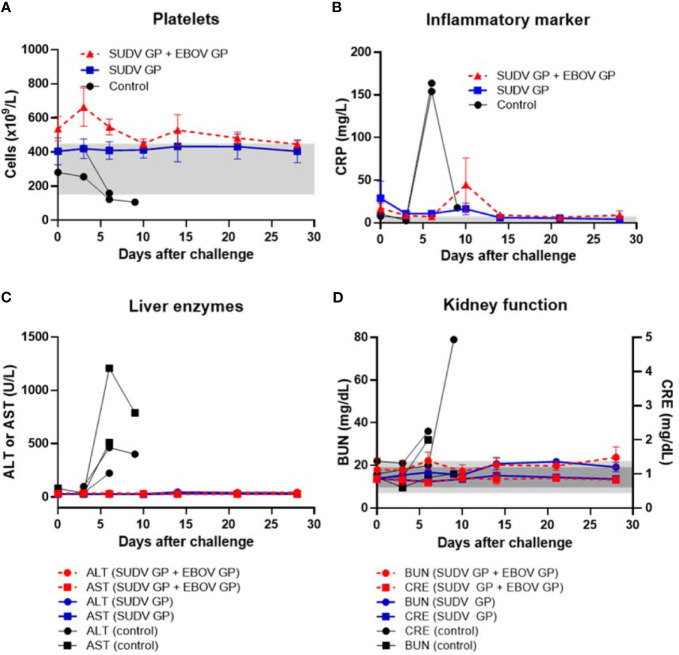
Platelet counts, inflammatory marker, liver enzymes, and kidney function after SUDV challenge. **(A)** Platelet counts with normal range of 150-450 x 10^3^ cells/μL shown as light grey band. **(B)** Serum CRP with normal range of 0.0-7.5 mg/L shown as light grey band. **(C)** Serum ALT with normal range of 10-47 U/L and serum AST with normal range of 11-38 U/L. **(D)** Serum BUN with normal range of 7-22 mg/dL shown as light grey band and CRE with normal range of 0.6-1.2 mg/dL shown as darker grey band. Each data point for vaccinated groups represents mean ± SEM. Data points for controls were plotted per individual animal.

After challenge with SUDV, all animals vaccinated with either formulation survived while all control animals succumbed to Sudan virus disease (SVD) by day 9 after viral challenge (**Figure 4C**). Control animals manifested increasing SUDV viremia after viral challenge compared to vaccinated groups that remained free from disease (**Figure 4D**). Similar to controls in the EBOV challenge, controls that succumbed to SVD after SUDV challenge also exhibited notable declines in platelet counts with concurrent surges in serum CRP, ALT, AST, BUN and CRE (**Figures 5A–D**).

**Figure 6:**
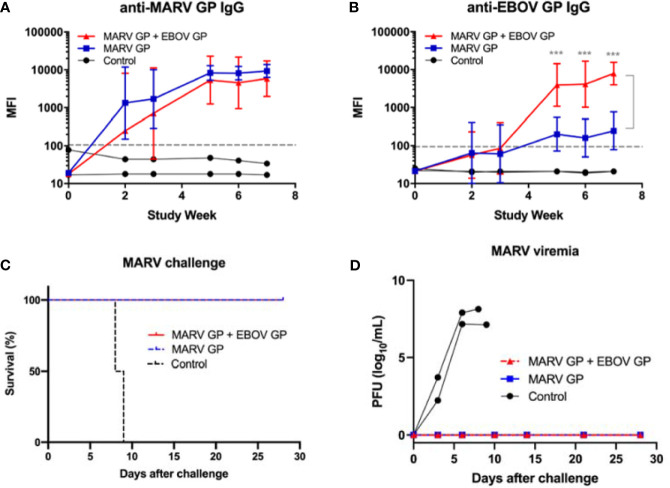
Immune response to MARV vaccination, survival and viremia after MARV challenge. MIA performed in triplicate for animals vaccinated at three-week intervals with three doses of MARV GP with adjuvant (n=4) or MARV GP + EBOV GP with adjuvant (n=4). Controls received adjuvant only (n=2): **(A)** MARV GP-binding IgG and **(B)** EBOV GP-binding IgG. Each data point for vaccinated groups represents geometric mean ± 95% confidence interval whereas data points for controls were plotted per individual animal. The dashed grey line marks the threshold for positive detection of antigen-specific IgG at 3 SD above mean MFI of negative control samples. **(C)** Kaplan-Meier survival curves after challenge with MARV. **(D)** MARV viremia as assessed by plaque assay. Each data point for vaccinated groups represents mean ± SEM. Data points for controls were plotted per individual animal. ***p < 0.001.

In an additional study, cynomolgus macaques were vaccinated with three doses of either 25 μg of MARV GP with adjuvant (n=4) or 25 μg of MARV GP + 25 μg of EBOV GP with adjuvant (n=4) to determine whether a different combination of filovirus vaccines would also retain efficacy. Controls received adjuvant only (n=2). Animals were challenged intramuscularly with 1,000 PFU of a low-passage, human isolate of MARV (Angola strain) at week 10 after initial vaccination. MARV GP-binding IgG levels increased for both vaccinated groups and from week three to the end of the study did not statistically differ between vaccine formulations (p>0.05) (**Figure 6A**). As expected, EBOV GP-binding IgG levels were higher in the group vaccinated with MARV GP + EBOV GP compared to the MARV GP group. This difference was statistically significant at each time point after the second vaccine dose (p<0.001) (**Figure 6B**).

**Figure 7:**
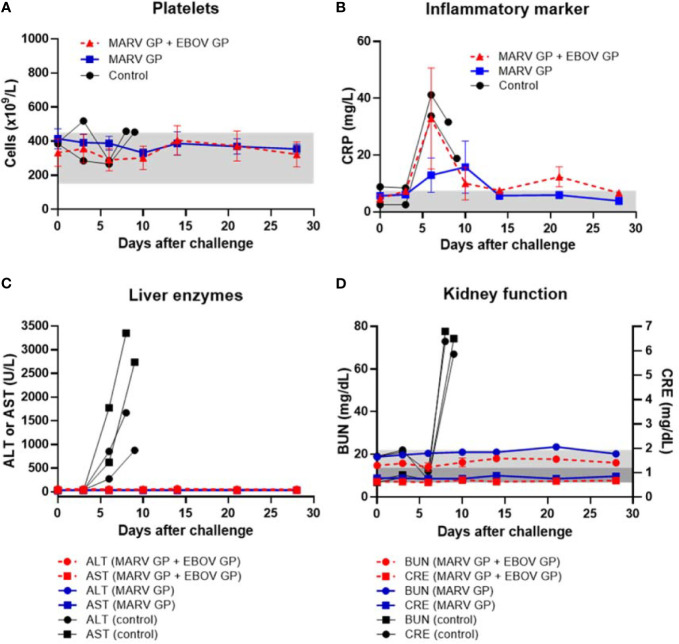
Platelet counts, inflammatory marker, liver enzymes, and kidney function after MARV challenge. **(A)** Platelet counts with normal range of 150-450 x 10^3^ cells/μL shown as light grey band. **(B)** Serum CRP with normal range of 0.0-7.5 mg/L shown as light grey band. **(C)** Serum ALT with normal range of 10-47 U/L and serum AST with normal range of 11-38 U/L. **(D)** Serum BUN with normal range of 7-22 mg/dL shown as light grey band and CRE with normal range of 0.6-1.2 mg/dL shown as darker grey band. Each data point for vaccinated groups represents mean ± SEM. Data points for controls were plotted per individual animal.

After challenge with MARV, all animals vaccinated with either formulation survived while all control animals succumbed to Marburg virus disease (MVD) by day 9 after viral challenge (**Figure 6C**). Control animals manifested increasing viremia after MARV challenge whereas vaccinated groups remained healthy and exhibited no MARV viremia (**Figure 6D**). Although their platelet counts did not drop, control animals challenged with MARV displayed increasing serum CRP, ALT, AST, BUN and CRE similar to that seen in controls in EBOV and SUDV challenge studies (**Figures 7A–D**).

## Discussion

While we have previously published data showing immunogenicity and protective efficacy of recombinant protein EBOV vaccines in mice and guinea pigs, we describe here for the first time that this vaccine platform successfully elicits potent antibody titers against EBOV, SUDV and MARV and protects cynomolgus macaques, a “gold standard” model in testing filovirus vaccines against viral challenge (15, 16). Cynomolgus macaques show uniform lethality when challenged with low passage human filovirus isolates. Similar to human EVD cases, animals develop fever within 3 days and experience viremia by day 5 post infection with EBOV (17). Cynomolgus macaques also develop clinical parameters of EVD such as high viremia, experience severe drops in platelet count, spikes in inflammatory markers such as C-reactive protein, and exhibit signs of liver and kidney toxicity as measured by elevated alanine aminotransferase, aspartate amino transferase, creatinine, and blood urea nitrogen (18, 19). Cynomolgus macaques also exhibit signs and symptoms similar to humans infected with the Gulu strain of *Sudan ebolavirus* (20) as well as *Marburg marburgvirus* (21).

Uniform protection against viral challenge was observed in all studies among all animals immunized with three doses of either monovalent or bivalent formulations. With limited filovirus cross-reactive immune response to monovalent MARV GP vaccine, our results suggest the feasibility of formulating a true multivalent filovirus vaccine with recombinant proteins protecting against the three most commonly occurring filoviruses pathogenic to humans. Furthermore, three out of four cynomolgus macaques vaccinated with just two doses of EBOV GP were fully protected against lethal challenge seven weeks after the last vaccination, demonstrating its prospective use as a safe and potent vaccine for outbreak response, such as a ring vaccination strategy around persons for whom Ervebo is contraindicated or for protection of healthcare workers entering areas of high-risk exposure. Although one cynomolgus macaque in the two-dose group did show signs of EVD, its onset of viremia as well as declining liver and kidney function was delayed compared to control animals. In a human outbreak, delaying onset of EVD signs and symptoms by even a few days could provide infected patients with essential time after viral exposure to seek care and receive treatment. However, we anticipate that the primary mode of deployment of thermostabilized recombinant subunit vaccines would be as a stockpiled prophylactic to be distributed to those at highest risk of exposure to filovirus infection, such as health care workers, civil and military first responders, and social service workers who may be in direct contact with potentially infected individuals. Thus, a prescribed three dose regimen would be practical as a prophylactic in at-risk populations; nevertheless, evidence suggests that there is rapid recall after an additional booster vaccination (data not shown), which would allow for a rapid and high degree of protection in pre-vaccinated individuals within days in the event of an outbreak. Furthermore, as both EBOV vaccines licensed to date employ the EBOV GP as their protective antigen and durability of immunity is a concern for both platforms, an EBOV GP protein vaccine can also be used to boost immunity in prior recipients of other EBOV vaccines.

Although other vaccine platforms targeting filoviruses have reached the clinical trial stage, virally vectored vaccines such as Ervebo and Ad26.ZEBOV/MVA-BN-Filo have cold chain requirements that make distribution and storage challenging in endemic regions with rudimentary infrastructure (22, 23). Recombinant protein vaccines offer safer, thermostable alternatives to virally vectored vaccines (24, 25). In prior publications, we showed that lyophilized component antigens and adjuvant as well as lyophilized complete vaccine formulations were highly stable at 40°C for 12 weeks, maintaining immunogenicity after extended periods at elevated temperatures (7, 11). Such thermostability offers a distinct advantage with regard to rapid distribution and stockpiling of vaccines in tropical regions where filovirus outbreaks have historically occurred.

In addition to the challenge of cold chain requirements, replication competent viral vaccines are contraindicated for severely immunocompromised individuals and pregnant women. Recommendations issued by the United States Advisory Committee on Immunization Practices (US ACIP) caution against immunizing these vulnerable groups with vaccines comprised of live virus (8). This issue is of particular concern in sub-Saharan Africa where filovirus outbreaks have occurred and the prevalence of human immunodeficiency virus (HIV) infection remains high (26). In central and west Africa only 47% of people living with HIV were virally suppressed in 2019 (27). Nearly 5 million individuals in Ebola endemic regions are outright not candidates for immunization with Ervebo. In the case of immune deficiencies, both primary and secondary immunodeficiencies may present an increased risk for both vaccine failure as well as adverse events due to uncontrolled replication of a live attenuated vaccine. In the case of HIV infection, the lack of competent T helper cells can result in an inability to control the vaccine virus replication and result in uncontrolled replication. This has been demonstrated in the accidental immunization of an HIV positive individual with a smallpox vaccine, in which the patient’s HIV status was unknown and the vaccine strain of the virus induced a severe vaccinia infection (28). The high disease burden of HIV paired with the population of otherwise ineligible persons demonstrates that there is a need for a vaccine platform which can be safely administered to these populations in order to practice disease prevention and outbreak intervention. The potential for disease transmission during the convalescent period through either breastmilk or seminal fluids poses a potential risk of propagating disease through households and to individuals who are not protected by vaccination. In an investigation regarding the recovery of virus from various clinical samples, EBOV was both recovered by culture and detected by RT-PCR in breast milk and semen, indicating a potential risk of mother to child transmission as well as sexual transmission. These vulnerable populations may be at high risk for complications associated with live attenuated (LAV), or otherwise replication competent viral vaccine platforms. For instance, cases of infant infection with LAV vaccine strains have been reported for vaccinia and rubella vaccination (29). Recombinant protein vaccines may offer a safer alternative for these populations. Studies regarding protein subunit influenza immunization have determined that while the immune response is distinctly different during pregnancy, protective efficacy can be achieved in this population through a protein subunit vaccine platform (30). In addition to enhancing protective efficacy, oil in water and alum adjuvants have been shown to be safe for administration during pregnancy (31), suggesting that a protein subunit platform adjuvanted with an oil in water emulsion adjuvant such as the candidate described here, may be both safe and efficacious, further enhancing the disease intervention methods already implemented in EVD endemic regions.

The 2013-2016 epidemic in West Africa demonstrated the pandemic potential of filoviruses with infected individuals crossing continents and oceans, given the great ease and speed of modern air travel (32). Even more recent outbreaks in DRC suggest the possibility of future filovirus pandemics. In addition to potential pandemic prevention, reduction of the burden of various long-term sequelae is key for overall community health. Individuals recovering from EVD experience various complications ranging from uveitis, myalgia, arthralgia, retroorbital pain, and mental-emotional disturbances such as depression and post-traumatic stress disorder. PTSD has been documented in both EVD survivors as well as uninfected survivors of the Ebola epidemic (33). Orphaned children face a very different form of childhood trauma marked by both negative Ebola stereotypes as well as the loss of caretakers (34). These mental and physical consequences of EVD, as well as other filovirus diseases, are pervasive but with widespread immunization and rapid distribution of vaccines, they can potentially be prevented. In summary, the recombinant protein vaccine platform described herein offers a safer, thermostable, and potentially multivalent alternative to virally vectored vaccines such as Ervebo – an additional defense to prevent future filovirus epidemics.

## Data Availability Statement 

The raw data supporting the conclusions of this article will be made available by the authors, without undue reservation.

## Ethics Statement 

The animal study was reviewed and approved by BIOQUAL and UTMB Institutional Animal Care and Use Committees.

## Author Contributions

AL and ML conceived and designed the studies and methodologies described. AL acquired funding and served as project administrator. Additional resources and supervision were provided by AL, HA, and TG. JY-O, JM, and AG managed NHP vaccinations, blood collections and processing prior to viral challenge. TW, EC, MN, and CW performed Luminex assays to document immunogenicity. JG, KA, RC, and TG conducted viral challenges of NHP and plaque assays, monitored pathogenesis and provided post-challenge haematology and chemistry results. AL, ML, and EC curated data. AL, ML, EC, and CW conducted formal analysis of data and drafted the manuscript. All authors contributed to the article and approved the submitted version.

## Conflict of Interest

AG, JM, JY-O and HAE were employed by BIOQUAL, Inc.

The remaining authors declare that the research was conducted in the absence of any commercial or financial relationships that could be construed as a potential conflict of interest.

## Publisher’s Note

All claims expressed in this article are solely those of the authors and do not necessarily represent those of their affiliated organizations, or those of the publisher, the editors and the reviewers. Any product that may be evaluated in this article, or claim that may be made by its manufacturer, is not guaranteed or endorsed by the publisher.
